# Statin Use is Associated with Decreased Hepatocellular Carcinoma Recurrence in Liver Transplant Patients

**DOI:** 10.1038/s41598-018-38110-4

**Published:** 2019-02-06

**Authors:** Yongin Cho, Myoung Soo Kim, Chung Mo Nam, Eun Seok Kang

**Affiliations:** 1 Division of Endocrinology and Metabolism, Department of Internal Medicine, Yonsei University College of Medicine, Seoul, Republic of Korea; 2 Department of Transplantation Surgery, Severance Hospital, Yonsei University Health System, Seoul, Republic of Korea; 3 Department of Preventive Medicine, Yonsei University College of Medicine, Seoul, Republic of Korea; 4 Institute of Health Services Research, Yonsei University College of Medicine, Seoul, Republic of Korea; 5 Institute of Endocrine Research, Yonsei University College of Medicine, Seoul, Republic of Korea

## Abstract

Statins have been reported to prevent the development of hepatocellular carcinoma (HCC). We examined whether statin therapy is associated with decreased HCC recurrence in patients who underwent liver transplantation for HCC. Three hundred forty-seven patients ≥ 20 years old who underwent liver transplantation for HCC from 2006 to 2016 were enrolled in this study. Statin therapy was defined as the administration of statins for more than 30 days after liver transplantation. One hundred twelve (32.3%) patients treated with statins over 30 days were defined as the statin group, and the remaining 235 (67.7%) were defined as the non-statin group. Several risk factors reported to be associated with HCC recurrence, such as proportion of underlying liver disease, above Milan criteria, differentiation of HCC, vascular invasion, and preoperative alpha-fetoprotein level were not different between the two groups. Time-dependent Cox regression analysis showed that statin treatment was associated with significantly lower recurrence risk of HCC after adjusting for other risk factors (hazard ratio = 0.32, 95% CI = 0.11–0.89).

## Introduction

Hepatocellular carcinoma (HCC) is a disease that continues to rise in frequency around the world. It is the second leading cause of cancer-related death in men and the sixth in women^1^. HCC occurs frequently in East and South-East Asia and in middle and western Africa, due to the high prevalence of chronic hepatitis B virus infection^2^. The prevalence of HCC in Europe and the United States is also expected to increase owing to the recent increase in patients with non-alcoholic fatty liver disease or chronic hepatitis C virus infection^3,4^.

Although surgical resection is considered to be the first treatment option for early-stage HCC, liver transplantation (LT) has shown better outcome than resection of the liver^5^. Moreover, it is expected that LT can not only remove the tumor, but also cure the underlying liver disease. Therefore, LT is regarded as one of the major treatment options for HCC^6^. Although the 5-year survival rate of HCC patients treated with LT has steadily improved^7^, the high recurrence rate of HCC after LT, estimated between 15% and 20%, is still an important clinical challenge^8^.

Statins, 3-hydroxy-3-methylglutaryl coenzyme A reductase inhibitors, are lipid-lowering agents that prevent cardiovascular disease and its related mortality^9^. Several previous randomized controlled trials monitoring statin usage showed the promising result of cancer prevention by statins in colorectal, prostate, breast, and skin cancer^10,11^. Statins also showed a remarkably favorable overall safety profile for long-term use in cancer prevention^12^. In addition, studies on the preventive effect of statins on HCC recurrence has also been published. Statin have been shown to contribute to the prevention of HCC development in hepatitis B patients^13,14^, hepatitis C patients^15,16^, patients who underwent initial liver resection due to HCC^17^, and the general population in a large cohort study^18^.

However, the effect of statins on HCC recurrence in patients who had LT for HCC has not been studied. When analyzing the effects of drugs on specific events in the observation study, immortal-time bias and selection bias often occur. Therefore, the approach to this should be done carefully; and for addressing the potential impacts of such bias, time-dependent exposure assignment methods will be needed. In this study, we investigated whether postoperative statin use affects the recurrence rate of HCC in patients who underwent LT for HCC.

## Results

### Baseline characteristics of the patients

The baseline characteristics are shown in Table 1. Among 347 patients, 114 patients had a history of statin use during the observation period, and 112 maintained statin use for more than 1 month (defined as the statin group). A total of 104 patients maintained statin use for more than 3 months. The median follow-up period of patients without recurrence was 44.9 (24.4–77.2) months, and the median statin administration period after LT was 22.7 (11.5–43.8) months.

**Table 1:** Baseline clinical characteristics of the patients.

	Non-statin group (n = 235)	Statin group (n = 112)	*p*-value
Age at the time of operation (y)	54.6 ± 7.0	56.7 ± 6.0	0.008
Sex (Female, %)	36 (15.3)	25 (22.3)	0.109
Liver allograft			0.090
Whole cadaver	63 (26.8)	40 (35.7)	
Living donor	172 (70.5)	72 (64.3)	
Underlying liver disease			0.164
Hepatitis B	198 (84.3)	86 (76.8)	
Hepatitis C	16 (6.8)	14 (12.5)	
non-viral	21 (8.9)	12 (10.7)	
Therapies for primary tumor control before operation	170 (72.3)	75 (67.0)	0.304
AFP (pre-op., ng/mL)	109.1 ± 453.3	62.4 ± 272.3	0.315
AFP over 50 ng/mL	51 (21.7)	17 (15.2)	0.152
PIVKA-II (pre-op., ng/mL)	300.2 ± 2214.0	112.0 ± 317.1	0.372
PIVKA-II over 50 ng/mL	91 (38.7)	32 (28.6)	0.065
[Post transplantation data]			
Number of tumors	3.3 ± 4.1	2.7 ± 2.3	0.149
Tumor size (Largest, cm)	2.7 ± 1.6	2.7 ± 1.7	0.882
Tumor size (Sum, viable, cm)	5.6 ± 5.5	4.8 ± 4.0	0.197
Above Milan criteria	102 (45.9)	46 (41.4)	0.436
Differentiation (Ed’s Grade)			0.584
I	26 (12.9)	16 (17.6)	
II	101 (50.2)	44 (48.4)	
III	72 (35.8)	31 (34.1)	
IV	2 (1.0)	0 (0.0)	
Differentiation (Ed’s Grade, well vs poor)			0.525
Well (I & II)	127 (63.2)	61 (67.0)	
Poor (III & IV)	74 (36.8)	30 (33.0)	
Microvascular invasion	61 (26.6)	19 (17.4)	0.063
Portal vein invasion or thrombosis	28 (12.1)	14 (12.7)	0.862
Anti-viral therapy after operation	170 (72.3)	75 (67.0)	0.304
Use of tacrolimus	235 (100.0)	111 (99.1)	0.147
Use of sirolimus	9 (3.8)	5 (4.5)	0.779
Use of cyclosporine	1 (0.4)	4 (3.6)	0.022
F/U Duration (months)	50.6 ± 36.9	55.9 ± 37.1	0.213
Death (Cancer unrelated)	20 (8.5)	9 (8.0)	0.881

Data are expressed as the mean ± SD for continuous variables and number (%) for categorical variables.

Abbreviations: AFP, alpha-fetoprotein; op., operation; PIVKA-II, prothrombin induced by vitamin K absence-II; Ed’s, Edmondson’s; F/U, Follow-up; HCC, Hepatocellular carcinoma.

The baseline characteristics of the patients and the histopathological factors confirmed immediately after the operation were compared between the two groups (statin group vs. non-statin group). Compared to the non-statin group, patients in the statin group were older (54.6 ± 7.0 years vs. 56.7 ± 6.0 years, *p* = 0.008). The proportion of female patients (15.3% vs. 22.3%, *p* = 0.109) and patients who received a liver allograft from a whole cadaver (26.8% vs. 35.7%, *p* = 0.090) were higher in the statin group without statistical significance. Other variables known to be associated with HCC recurrence, such as the proportions of underlying liver disease, above Milan criteria, differentiation of HCC, microvascular invasion, portal vein invasion or thrombosis, and preoperative AFP level, were not significantly different between the two groups.

### Factors associated with tumor recurrenc**e**

In univariate analysis, younger age (odds ratio [OR] = 0.94, 95% CI = 0.90–0.98), number of tumors (OR = 1.11, 95% CI = 1.02–1.21), largest tumor size (OR = 1.30, 95% CI = 1.11–1.52), sum size of viable tumors (OR = 1.08, 95% CI = 1.02–1.14), proportion of subjects who met “above Milan criteria” (OR = 2.91, 95% CI = 1.62–5.22), poor differentiation based on Edmondson grade (OR = 2.40, 95% CI = 1.31–4.40), microvascular invasion (OR = 4.74, 95% CI = 2.61–8.59), portal vein invasion or thrombosis (OR = 4.74, 95% CI = 2.37–9.49), and preoperative AFP over 50 ng/mL (OR = 5.08, 95% CI = 2.76–9.35) were significantly associated with increased recurrence rate, similar to what has been previously reported (Table 2).

**Table 2:** Characteristics of HCC cases with and without recurrence.

	Without Recurrence	With Recurrence	Crude OR
	(n = 288)	(n = 59)	(95% CI)
Age at the time of operation (y)	55.7 ± 6.7	52.9 ± 6.6	0.94 (0.90–0.98)
Sex (Female, %)	51 (17.8)	8 (13.1)	0.70 (0.31–1.55)
Liver allograft			
Whole cadaver	87 (30.2)	16 (27.1)	1.00
Living donor	201 (69.8)	43 (72.9)	1.16 (0.62–2.18)
Underlying liver disease			
non-viral	31 (10.8)	2 (3.4)	1.00
Hepatitis B	232 (80.6)	52 (88.1)	3.47 (0.81–14.98)
Hepatitis C	25 (8.7)	5 (8.5)	3.10 (0.55–17.35)
Number of tumors	2.9 ± 2.6	4.4 ± 6.5	1.11 (1.02–1.21)
Tumor size (Largest, viable, cm)	2.5 ± 1.6	3.3 ± 1.8	1.30 (1.11–1.52)
Tumor size (Sum, viable, cm)	4.9 ± 4.2	7.3 ± 7.9	1.08 (1.02–1.14)
Above Milan Criteria	111 (39.8)	37 (68.5)	2.91 (1.62–5.22)
Differentiation (Ed’s Grade)			
I	42 (17.6)	0 (0.0)	1.00
II	120 (50.2)	25 (47.2)	uncheckable
III	77 (32.2)	26 (49.1)	uncheckable
IV	0 (0.0)	2 (3.8)	uncheckable
Differentiation (Ed’s Grade, well vs poor)			
Well (I, II)	163 (68.2)	25 (47.2)	1.00
Poor (III, IV)	76 (31.8)	28 (52.8)	2.40 (1.31–4.40)
Microvascular invasion	50 (17.9)	30 (50.8)	4.74 (2.61–8.59)
Portal vein invasion or thrombosis	24 (8.5)	18 (30.5)	4.74 (2.37–9.49)
AFP (pre-op., ng/mL)	46.6 ± 186.6	325.7 ± 857.8	1.002 (1.000–1.003)
AFP over 50 ng/mL	41 (14.2)	27 (45.8)	5.08 (2.76–9.35)
PIVKA-II (pre-op., ng/mL)	140.0 ± 1000.8	724.7 ± 3843.2	1.000 (1.000–1.000)
PIVKA-II over 50 ng/mL	85 (29.5)	38 (64.4)	4.32 (2.40–7.80)
Anti-viral therapy after operation	201 (69.8)	44 (74.6)	1.27 (0.67–2.40)

Data are expressed as the mean ± SD for continuous variables and number (%) for categorical variables.

Abbreviations: HCC, hepatocellular carcinoma; OR, odd ratio; CI, confidence interval; Ed’s, Edmondson’s; AFP, alpha-fetoprotein; op., operation; PIVKA-II, prothrombin induced by vitamin K absence-II.

The factors affecting recurrence of HCC were compared by multiple logistic regression model. In variant-adjusted models, statin therapy was associated with a reduced risk of HCC recurrence (aOR = 0.38, 95% CI = 0.16–0.91; Supplementary Table 1). When the cumulative periods of statins were divided by tertile, long-term statin use (≥485 days; middle and high tertiles) showed a significantly protective role against HCC recurrence, even after adjusting for multiple confounding factors.

### Time-dependent analysis

**Figure 1:**
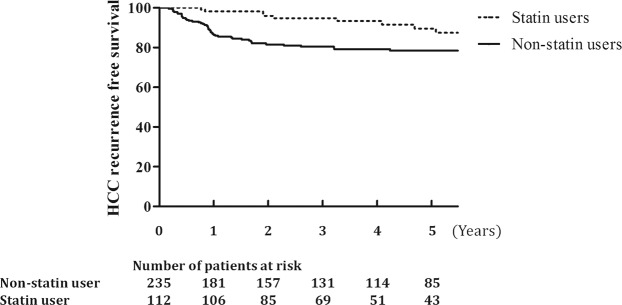
Kaplan-Meier curve of HCC recurrence-free survival. Dotted line, statin group (>1 month use); solid line, non-statin group.

Statin use longer than 1 month showed significant benefit for preventing HCC recurrence by Kaplan-Meier curve (Fig. 1). However, the proportional hazards assumption was not valid on the validity approaches for using stepwise Cox regression analysis. Except in patients with preexisting dyslipidemia, statin therapy was not started immediately after surgery. Since the initiation time of statin use was different within the statin group, a bias may have been present in this process. To minimize this bias, time-dependent Cox regression analysis was performed. Crude HR results were obtained by setting the lag period to 1, 2, 3, 6, and 12 months (Supplementary Figure 1), and considering the follow-up interval of examinations and outpatient clinic, patients who maintained statin for more than 3 months (1 month of exposure and 2 months of lag period) were assigned to the statin treatment group in this analysis. The result showed that statin therapy was significantly related to lower recurrence rates, even after adjusting for the initiation time of statins and other factors that showed relationships with HCC recurrence in univariate analysis (hazard ratio (HR) = 0.32, 95% CI = 0.11–0.89). Other risk factors showed similar results with those of the logistic regression analysis (Table 3).

**Table 3:** Independent predictors of HCC recurrence in LT patients using time-dependent Cox regression analysis.

		Model 1	Model 2
	Crude HR	Adjusted HR*	Adjusted HR**
	(95% CI)	(95% CI)	(95% CI)
Age at the time of operation (y)	0.96 (0.92–0.99)		0.98 (0.94–1.02)
Sex (Female)	0.75 (0.35–1.57)		1.04 (0.46–2.35)
Liver allograft (Living donor)	1.08 (0.61–1.92)		
Underlying liver disease			
Non-viral	1.00		
Hepatitis B	2.69 (0.66–11.07)		
Hepatitis C	2.40 (0.47–12.39)		
Anti-viral therapy after operation	1.27 (0.67–2.40)		
Number of tumors	1.08 (1.04–1.12)		
Tumor size (Largest, Viable)	1.25 (1.11–1.40)		
Tumor size (Sum, Viable)	1.07 (1.03–1.10)		
Above Milan Criteria	2.78 (1.63–4.74)	2.51 (1.41–4.49)	2.55 (1.43–4.56)
Differentiation			
Poor (Ed’s Grade III, IV)	2.52 (1.47–4.34)	1.72 (0.97–3.07)	1.63 (0.91–2.95)
Microvascular invasion	4.04 (2.42–6.74)		
Portal vein invasion or thrombosis	4.20 (2.40–7.34)	3.42 (1.84–6.38)	3.33 (1.77–6.27)
AFP (pre-op., ng/mL)	1.001 (1.000–1.001)		
(AFP over 50 ng/mL)	4.29 (2.57–7.16)	3.40 (1.97–5.87)	3.24 (1.86–5.65)
PIVKA-II (pre-op., ng/mL)	1.000 (1.000–1.000)		
(PIVKA-II over 50 ng/mL)	3.76 (2.21–6.41)		
Statin users (time dependent, over 3 months)	0.32 (0.11–0.88)	0.30 (0.11–0.83)	0.32 (0.11–0.89)

*Model 1 adjusted for Above Milan Criteria, grade (High vs Low), portal vein invasion or thrombosis, and preoperative alpha fetoprotein > 50 ng/mL; **Model 2 adjusted for model 1 parameters + age and sex

Abbreviations: HCC, hepatocellular carcinoma; LT, liver transplantation; HR, hazard ratio; CI, confidence interval; Ed’s, Edmondson’s; AFP, alpha-fetoprotein; op., operation; PIVKA-II, prothrombin induced by vitamin K absence-II.

Time-dependent Cox analysis was also performed by changing the outcome to mortality without applying the lag period. The results were similar to those obtained when the outcome was tumor recurrence, showing significant differences only in cancer-related mortality (HR = 0.30, 95% CI = 0.09–0.97, Supplementary Table 2). However, there was a limitation in mortality analysis since the mortality information in this study database was unclear, and there was more room for bias to occur.

### Subgroup analysis

Using classification according to the criteria based on explant pathology, we divided the entire patient group into “Above Milan” group and others, and proceeded with subgroup analysis (Supplementary Figure 2). Time-dependent Cox analysis was performed in each group. Crude HR (time-dependent) was 0.24 (0.06–0.99, p = 0.048) in “Above Milan” group; and in the remaining subjects, crude HR was 0.45 (0.11–1.96, p = 0.290, Supplementary Table 3).

Including seven cases who were confirmed to have been used statins before liver transplantation, 64 cases started statins within 1 year after liver transplantation and 48 cases started after 1 year of LT. After dividing the statin groups according to the start point of statins, time-dependent Cox regression analysis was performed with non-statin exposure groups for each group. When started statins within 1 year after transplantation, crude HR was 0.18 (0.04–0.75, p = 0.018) compared to non-statin group, whereas crude HR was 0.73 (0.17–3.19, p = 0.674) in statin groups who had longer than 1-year period to start statins after transplantation (Supplementary Table 3).

## Discussion

LT has several advantages for treating HCC, such as a relatively low recurrence rate and survival benefit compared to other options. Therefore, it is one of the most useful and important treatment methods, especially in patients with concern about liver dysfunction. However, the recurrence rate of HCC after LT, estimated between 15% and 20%, is still an important clinical issue for LT recipients^8^, and the 5-year survival was significantly lower in patients with recurrence (22%) than in patients without recurrence (64%)^19^.

In the present study, the use of statins was significantly associated with a lower HCC recurrence rate in LT recipients (aOR = 0.38, 95% CI = 0.16–0.91). This is consistent with results from previous studies, including a recent large-scale cohort analysis, showing that the use of statins reduced the incidence of HCC^13,15,18,20^. However, the longer survival period increases the opportunity for exposure to statins. For this reason, bias may occur in studies with a retrospective design. Furthermore, there is controversy about how long a period of statin therapy is required to assume a protective role against tumor recurrence. Therefore, in this study, time-dependent Cox analysis was performed by adjusting the known risk factors related to HCC recurrence. As a result, we could confirm that statins played a role in preventing HCC recurrence, even after adjusting for various risk factors.

Previous studies have shown that the effect of statins on decreasing the risk of HCC is more evident in high-risk patients, such as patients with chronic liver disease (aOR = 0.32, 95% CI = 0.17–0.57)^21^ or diabetes mellitus (aOR = 0.36, 95% CI = 0.22–0.60)^20^. Hepatic steatosis and chronic inflammation, which are prominent in conditions such as diabetes^22^ and liver disease, are also associated with hepatocellular carcinogenesis, and statins have a possible protective role by ameliorating these factors associated with chronic inflammation^23,24^. A similar degree of risk reduction with statin treatment was obtained in this study (aHR = 0.32, 95% CI = 0.11–0.89). This is probably related to exposure to high-risk conditions for HCC recurrence after LT. In subgroup analysis, patients who started statin early, as well as the high risk group that corresponded to the “Above Milan” criteria, showed more significant effect by statins. However, considering that subgroup analysis showed a decreased statistical power with shorter overall observation period, more patients and observation time will be needed to see more definite results.

The use of statin was also shown to be effective in improving cancer-related mortality; however, bias may have been greater due to the limitations of our study database and design. Therefore, any judgment made against it should be reserved. However, since recurrence of HCC leads to an increase in mortality, statin use may also contribute to decrease of mortality.

It is unclear which mechanism of statins contributes to the prevention of HCC. In a recent review article, the authors suggested some potential anticancer properties of statins. The inhibition of 3-hydroxy-3-methylglutaryl coenzyme A reductase by statins reduces mevalonate pathway metabolites that are essential for cancer cell growth and survival^25^. In this regard, potential mechanisms of statins, such as antiproliferative effects^26^, apoptosis induction^27^, and anti-invasive effects^28^, have been seen in many cancer types^29^. Although these studies cannot explain the direct effect of statins on HCC, it is assumed that statins can contribute to HCC prevention, given that the primary target organ of statins is the liver.

Other factors known to increase the recurrence rate after LT have been presented in previous reports. In a meta-analysis of HCC recurrence, patients who did not meet the Milan criteria (patients with solitary HCC of less than 5 cm or with up to three nodules of less than 3 cm)^5^ had worse overall and disease-free survival and experienced more recurrence after LT for HCC. Total tumor diameter, diameter of the largest tumor, and number of tumors were all associated with higher risk of HCC recurrence^30^. When evaluating preoperative tumor load, standard imaging methods such as liver CT can underestimate or overestimate the extent of HCC in up to 25% of cases, compared with pathological findings of the explanted liver^31^. For this reason, in this study, total tumor size, size of the largest tumor, and number of tumors were measured based on the pathologic findings. Moreover, these factors associated with preoperative tumor load were closely related to HCC recurrence in our study, similar to the results of previous studies^5,19,30^. We found that patients who met “above Milan criteria” were especially closely associated with increased risk of HCC recurrence after adjusting for other risk factors (HR = 2.55, 95% CI = 1.43–4.56).

Vascular invasion^32,33^ and histological grade^32^ of HCC also affect prognosis after LT. In our study, we also noted that microvascular invasion, portal vein invasion or thrombosis, and histological grade increased the risk of HCC recurrence. The preoperative AFP concentration was associated with tumor load, and elevated AFP was related to postoperative HCC recurrence in several previous studies^34^. In this study, high AFP concentration was classified as over 50 ng/mL^35^, and it was confirmed that high AFP concentration was related to high recurrence rate of HCC (HR = 3.24, 95% CI = 1.86–5.65), consistent with the results of previous studies. Another serum tumor marker, prothrombin induced by vitamin K absence-II, was also measured. Prothrombin induced by vitamin K absence-II has been reported to be associated with microvascular invasion^36,37^, and it was also associated with HCC recurrence rate, as well as AFP level, in our study (crude HR = 3.76, 95% CI = 2.21–6.41).

In several previous studies, the effect of graft type on tumor recurrence was unclear^38,39^. We found no difference in risk according to graft type in this study (crude HR = 1.08, 95% CI = 0.61–1.92). Additionally, some studies have found that overexposure to immunosuppressive agents such as tacrolimus is associated with high HCC recurrence risk^35^; however, there are no randomized controlled trials suggesting that lowering immunosuppressant concentration is related to lower risk of HCC recurrence. The postoperative anti-viral treatment did not show a significant effect on HCC recurrence, unlike the results of previous literature^40^. In our institution, the presence of chronic hepatitis was monitored regularly after transplantation, and anti-viral therapy was applied immediately after the sign of chronic viral hepatitis. Since anti-viral treatment was performed unconditionally in the patients corresponding to treatment indication, it was impossible to determine whether there was a difference in the occurrence of tumor recurrence according to anti-viral treatment. All of the factors that have been shown to affect recurrence of HCC were included in final multivariate analysis in this study.

The recurrence of HCC after LT has great influence on various vital aspects, such as survival rate and quality of life. However, an effective therapeutic option has not yet been established. Therefore, various efforts are underway for the prevention of HCC recurrence. There has been a wide range of attempts, including administration of adjuvant chemotherapy^41^, adjuvant oncolytic adenoviral therapy^42^, the heparinase inhibitor PI-88^43^, interferon-alpha^44^, and sorafenib^45^. Several studies showed survival benefit associated with treatment; however, no treatment has been established as an effective universal method^8^ in the prevention of HCC recurrence.

Despite concerns about their diabetogenic effect, statins are effective and safe for the prevention of cardiovascular disease in patients with dyslipidemia, and are often used in LT recipients^46^. Although there is a lack of research regarding statin therapy in LT recipients, statin therapy may be an attractive, low-risk treatment option.

There are several limitations of this study. This was a retrospectively designed study, and thus, the duration of statin maintenance, follow-up period, and statin initiation time were different among the study population. At baseline conditions, the statin group showed lower AFP and higher differentiation level compared to the non-statin group, although it was not statistically significant. In addition, considering the limitation of this study design, clinical trial will be needed for statins to be used as a HCC therapeutic option. Second, in this study, statin effect may be overestimated or underestimated since the number of patients using statin was not large enough (considering follow-up period) and the number of events was relatively small.

However, the strength of this study is that we examined the statin effect after correcting the differences of these limits and conditions through time-dependent Cox analysis. In addition, since the study was performed in a single institution, conditions such as transplantation indication, surgery technique, and follow-up after the operation did not show much difference between patients. This study supports a large-scale observational study of statins’ role in the prevention of HCC development, and shows that use of statins in liver transplant recipients for the reduction of cardiovascular risk may also be helpful in preventing HCC recurrence.

In conclusion, statin use is associated with low HCC recurrence in LT recipients.

## Methods

### Study subjects

Seven hundred forty-seven patients received LT at the Severance Hospital from 2006 to 2016. Patients younger than 20 years of age and patients who had LT for cirrhosis without confirmed HCC were not included (n = 376). Twenty-three patients with early postoperative mortality (defined as death within 2 months after LT without signs of tumor recurrence) were excluded from the study. One patient with distant lymph node metastasis confirmed within 1 month after LT was also excluded. There were no postoperative statin users among the 24 excluded subjects. The remaining 347 patients were finally enrolled in this study.

Data about baseline characteristics, including allograft type and underlying liver disease, were collected. Preoperative tumor markers (alpha-fetoprotein (AFP) and prothrombin induced by vitamin K absence-II), postoperative pathology data (number and diameter of tumors), and follow-up examination results were also collected.

The group that did not meet the Milan criteria was included in the analysis as the single variable “above Milan criteria” based on the pathologic findings. In patients who underwent bridging treatments such as liver resection or transcatheter arterial chemoembolization, only identified viable tumors were included in the analysis, according to the European Association for the Study of the Liver guidelines^47^. The histologic differentiation of HCC (well (Grade 1–2) vs. poor (Grade 3-4)) was determined according to the Edmondson grade^48^.

Statin therapy was defined as postoperative statin use for more than 1 month. The cumulative doses of statin therapy were calculated as the sum of the statin doses during the treatment period and expressed as the cumulative defined daily dose according to the World Health Organization definition^49^. In the time-dependent analysis, patients who were treated with statin for 3 months or longer were defined as the exposed group to reduce bias on the test interval and the drug effect.

The Severance Hospital Institutional Review Board approved the study protocol (IRB No. 4-2017-0942), and patient consent was not necessary because of the retrospective chart review study design. In addition, all investigations were performed in accordance with the principles of the Declaration of Helsinki.

### Recipients of liver transplantation and postoperative follow up

Pre-transplant HCC workup routinely included liver contrast-enhanced computed tomography (CT), positron emission tomography, and/or chest CT. The extent of HCC lesions were routinely investigated before operation. Furthermore, patients were classified according to the Milan criteria based on preoperative imaging study findings. Candidates for LT were determined by using the Extended Milan criteria.

Before LT, a total of 245 patients (70.6%) underwent several therapies for primary tumor control including transcatheter arterial chemoembolization (n = 209, 60.2%), radiofrequency ablation (n = 69, 19.9%), surgical resection (n = 25, 7.2%), systemic chemotherapy (n = 15, 4.3%), or other therapies. Remaining 102 patients (29.4%) did not receive any anti-tumor treatment before transplantation, due to poor liver function, unspecific image findings, or because LT was imminently organized.

After LT, patients were again classified according to the Milan criteria based on explant pathology. Follow up liver CT was performed at 1 week, 1 month, 6 months, and every year until 5 years after surgery. Afterwards, liver CT was performed at 2-year intervals. Measurement of blood tumor markers [AFP and prothrombin induced by vitamin K absence-II] was performed every month for 6 months after discharge from the hospital and every 3 months thereafter. Additional liver CT, liver magnetic resonance imaging, or positron emission tomography-CT was performed when elevated tumor markers or suspicious lesions were noted. A new lesion with radiographic features compatible with HCC was defined as recurrence of HCC.

### Statistical analysis

All values are expressed as means ± standard deviations or numbers and percentages. The normality of the distribution of continuous variables was examined with Shapiro-Wilk tests. Between-group differences of mean values were compared with independent t-tests, and between-group differences of numbers and percentages were compared with χ^2^ tests.

Multiple logistic regression analysis was performed to evaluate the association between statin therapy and the risk of HCC recurrence after LT. Adjusted odds ratios (aORs) and 95% confidence intervals (CIs) were determined. Kaplan-Meier survival curves were used to calculate recurrence-free survival.

For stepwise Cox regression analysis, two approaches were used to assess the validity of the proportional hazards assumption. When assessing the validity by log-minus-log-survival function, it showed a vague aspect especially in the immediate postoperative period (Supplementary Figure 3). The time-dependent covariate was also statistically significant (p = 0.049), suggesting that the proportional hazards assumption was unreasonable. Since the patients did not start statin therapy at the same time point after LT, bias may have occurred. The time-dependent Cox model was used to consider this bias. Patients visited an outpatient clinic every 3 months, and tumor markers were followed up at each visit. Statins were mainly prescribed at the outpatient clinic at the same time. For this reason, patients who maintained statin use for more than 3 months were assigned to the statin treatment group in this analysis. Time-dependent Cox regression was performed with adjustments for significant predictors in the univariate analysis, as well as other factors previously known to be associated with the recurrence of HCC after LT.

*P*-values < 0.05 were considered statistically significant. All statistical analyses were performed with the Statistical Package for Social Sciences for Windows version 23.0 (SPSS, Inc., Chicago, IL, USA).

## Supplementary information

Dataset 1

## Data Availability

The datasets used and/or analysed during the current study are available from the corresponding author on reasonable request.
