# Supplementary material for: Statin Use is Associated with Decreased Hepatocellular Carcinoma Recurrence in Liver Transplant Patients
Source: Sci Rep. 2019 Feb 6;9:1467. doi: 10.1038/s41598-018-38110-4 (PMC6365496; doi:10.1038/s41598-018-38110-4)

Statin Use is Associated with Decreased Hepatocellular Carcinoma Recurrence in Liver Transplant Patients

Yongin Cho[1], Myoung Soo Kim[2], Chung Mo Nam[3,4], Eun Seok Kang[1.5]

[1]Division of Endocrinology and Metabolism, Department of Internal Medicine, Yonsei University College of Medicine, Seoul, Republic of Korea

[2]Department of Transplantation Surgery, Severance Hospital, Yonsei University Health System, Seoul, Republic of Korea

[3]Department of Preventive Medicine, Yonsei University College of Medicine, Seoul, Republic of Korea

[4]Institute of Health Services Research, Yonsei University College of Medicine, Seoul, Republic of Korea018

[5]Institute of Endocrine Research, Yonsei University College of Medicine, Seoul, Republic of Korea

Correspondence: Eun Seok Kang, MD, PhD

Division of Endocrinology and Metabolism

Department of Internal Medicine, Yonsei University College of Medicine, 50-1, Yonsei-ro, Seodaemun-gu, Seoul 03722, Republic of Korea

Telephone: +82 2 2228 1968, Fax: +82 2 393 6884, E-mail: edgo@yuhs.ac

Supplemental Table 1. Statin use in HCC cases with and without recurrence

				Model 1	Model 2	Model 3
	Without Recurrence (n = 288)	With Recurrence (n = 59)	Crude OR (95% CI)	Adjusted OR* (95% CI)	Adjusted OR** (95% CI)	Adjusted OR*** (95% CI)
Statin use						
Never use	185 (64.2)	50 (84.7)	1.00	1.00	1.00	1.00
Ever use (over 1 month)	103 (35.8)	9 (15.3)	0.32 (0.15-0.68)	0.36 (0.17-0.77)	0.37 (0.16-0.89)	0.38 (0.16-0.91)
Cumulative dose of use						
Never use	183 (64.0)	50 (84.7)	1.00	1.00	1.00	1.00
Ever use						
Tertile 1 (<485 cDDDs)	29 (10.1)	7 (11.9)	0.88 (0.37-2.14)	0.97 (0.40-2.36)	1.42 (0.49-4.09)	1.56 (0.54-4.54)
Tertile 2,3 (≥485 cDDDs)	74 (25.9)	2 (3.4)	0.10 (0.02-0.42)	0.11 (0.03-0.47)	0.10 (0.02-0.46)	0.09 (0.02-0.43)

*Model 1 adjusted for age and sex; **Model 2 adjusted for age, sex, Above Milan Criteria, grade (High vs Low), portal vein invasion or thrombosis, and preoperative alpha fetoprotein > 50 ng/mL; ***Model 3 adjusted for model 2 parameters + microvascular invasion, liver allograft, and underlying liver disease

Abbreviations: HCC, hepatocellular carcinoma; OR, odd ratio; CI, confidence interval; cDDDs, cumulative defined daily doses

Supplemental Table 2.

Independent predictors of cancer-related and all-cause mortality in LT patients using time-dependent Cox regression analysis

	Cancer-related	All-cause
	Crude HR (95% CI)	Crude HR (95% CI)
Age at the time of operation (y)	0.94 (0.90-0.99)	1.01 (0.98-1.05)
Sex (Female)	0.58 (0.21-1.63)	0.87 (0.44-1.70)
Liver allograft (Living donor)	0.87 (0.44-1.72)	0.59 (0.36-0.96)
Underlying liver disease		
Non-viral	1.00	1.00
Hepatitis B	3.26 (0.45-23.84)	0.71 (0.32-1.57)
Hepatitis C	0.88 0.06-14.16)	0.62 (0.20-1.95)
Anti-viral therapy after operation	1.58 (0.72-3.44)	0.77 (0.47-1.27)
Number of tumors	1.08 (1.03-1.13)	1.04 (0.99-1.10)
Tumor size (Largest, Viable)	1.34 (1.17-1.54)	1.19 (1.05-1.34)
Tumor size (Sum, Viable)	1.08 (1.04-1.12)	1.04 (1.00-1.08)
Above Milan Criteria	2.98 (1.52-5.83)	1.42 (0.88-2.30)
Differentiation		
Poor (Ed's Grade III, IV)	2.45 (1.26-4.76)	1.89 (1.13-3.16)
Microvascular invasion	5.54 (2.91-10.56)	2.91 (1.79-4.72)
Portal vein invasion or thrombosis	5.00 (2.50-9.99)	2.44 (1.33-4.50)
AFP (pre-op., ng/mL)	1.001 (1.000-1.001)	1.000 (1.000-1.001)
(AFP over 50 ng/mL)	5.45 (2.88-10.31)	3.03 (1.85-4.96)
PIVKA-II (pre-op., ng/mL)	1.000 (1.000-1.000)	1.000 (1.000-1.000)
(PIVKA-II over 50 ng/mL)	4.91 (2.43-9.89)	2.30 (1.42-3.71)
**Statin users (time dependent, without lag period)**	**0.30 (0.09-0.97)**	**0.80 (0.42-1.50)**

Abbreviations: HCC, hepatocellular carcinoma; LT, liver transplantation; HR, hazard ratio; CI, confidence interval; Ed's, Edmondson's; AFP, alpha-fetoprotein; op., operation; PIVKA-II, prothrombin induced by vitamin K absence-II.

Supplemental Table 3. Subgroup analysis according to the Milan criteria and start point of statins

	crude HR (95% CI) of statin use (time dependent, over 3 months)	p value
Above Milan criteria	**0.24 (0.16-0.99)**	**0.048**
Not above Milan criteria	**0.45 (0.11-1.96)**	**0.290**
Use of statin within 1 year of operation	**0.18 (0.04-0.75)**	**0.018**
Use of statin after 1 year of operation	**0.73 (0.17-3.19)**	**0.674**

Supplemental Figure 1. Trends in crude hazard ratios (HR, time dependent),with 95% confidence intervals (Cis), for the association between use of statins (exposure) and risk of HCC recurrence (outcome) according to lag times of increasing width

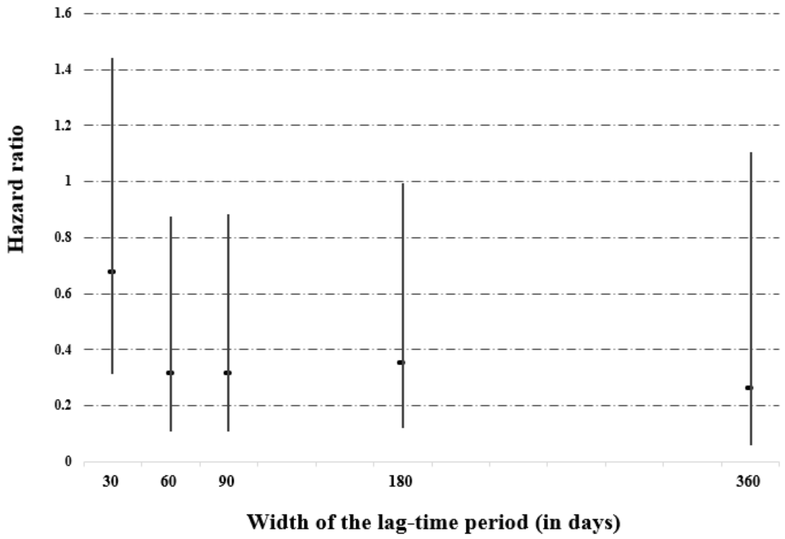

Supplemental Figure 2. Kaplan-Meier curve of HCC recurrence-free survival, subgroup analysis

Dotted line, statin group (> 1 month of use); solid line, non-statin group

(a). "Above Milan" group

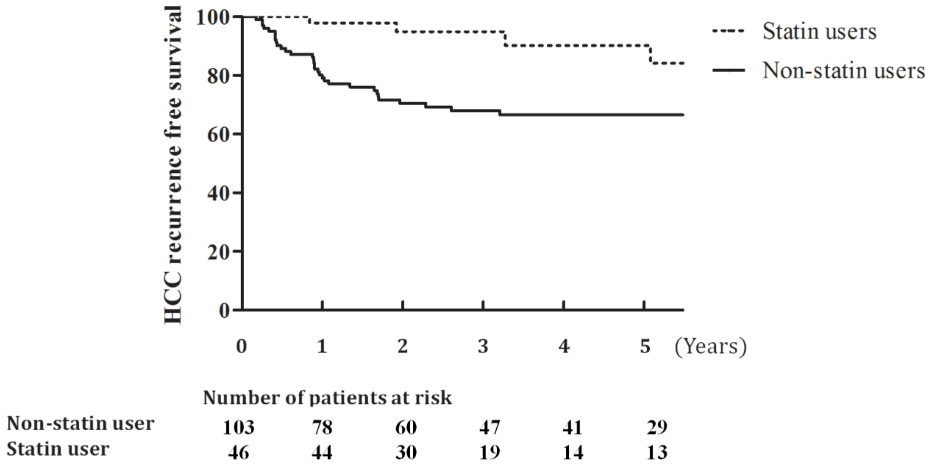

(b). "Not above Milan" group

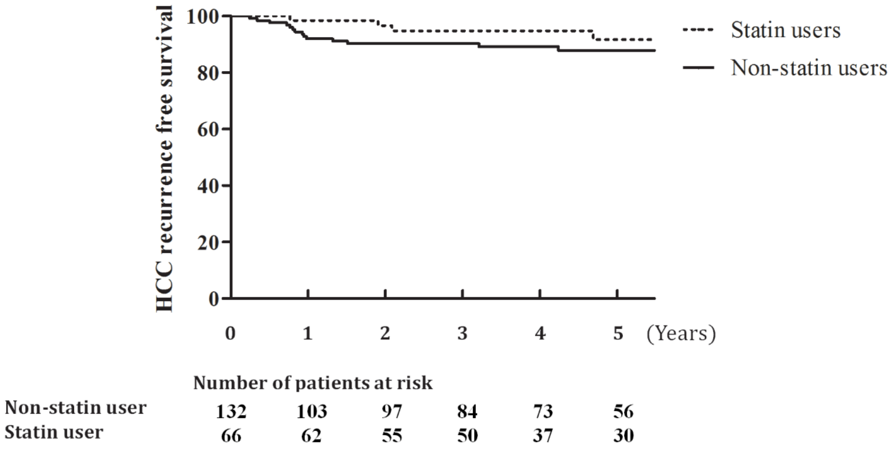

Supplemental Figure 3. Log-minus-log survival function graph against time separated by statin usage

Validity of proportional hazards assumption was assessed by log-minus-log-survival function. Dotted line, statin group (> 1 month); solid line, non-statin group

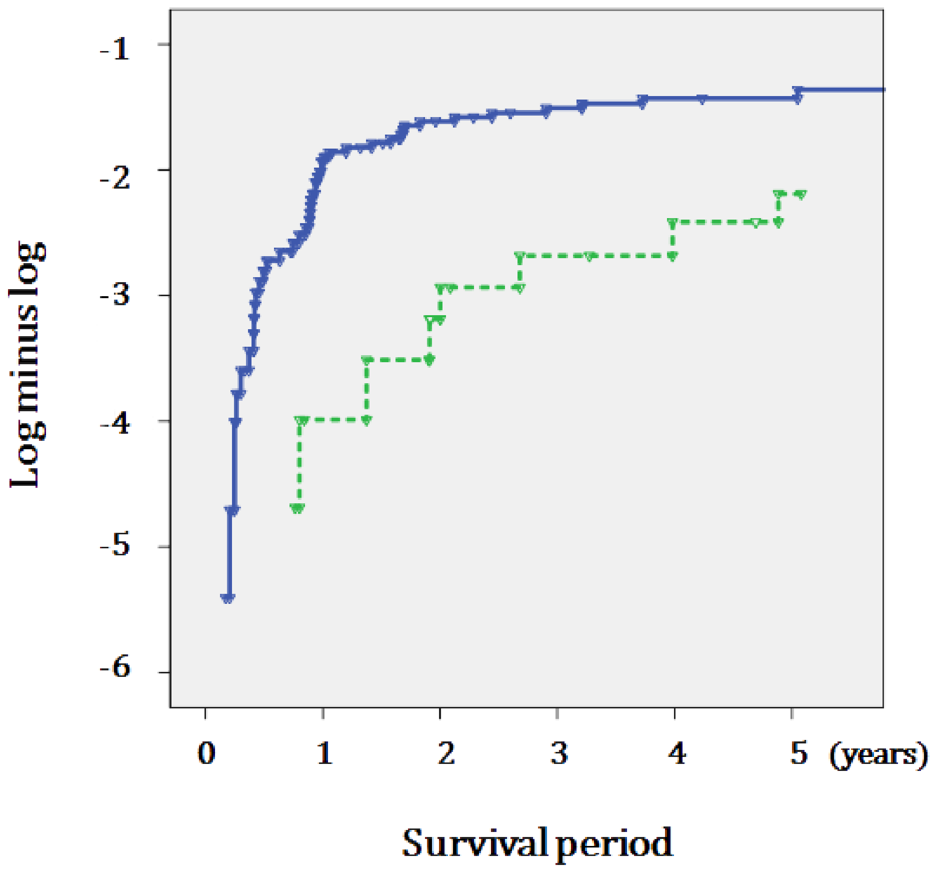

Supplement: Supplementary file 1 — Dataset 1 [file 41598_2018_38110_MOESM1_ESM.pdf]
